# The Recent Hospital Dispute

**Published:** 1889-12-14

**Authors:** 


### FALLOW CROPS.

### THE RECENT HOSPITAL DISPUTE.

In the interests of the institution chiefly concerned, as of hospitals generally, it is to be hoped the last has been heard of the late unfortunate squabble. The secretary has been "sacrificed," and the medical staff are vindicated. From the first it was to be expected that this would be the end of the controversy, and having had the misfortune to fall out both with his council and the staff, it might have been more polite, if less courageous, had Mr. Austin quietly accepted the inevitable. But when he was content in support of a very grave allegation to subpœna Mr. Æsculapius Scalpel as sole witness the proceedings ceased to be serious, except in so far as they affected his own position, and, instead of incurring a dignified martyrdom for the sake of truth, he made nothing so conspicuous as his worship of fiction.

Yet something more than a passing regret may be felt that one who seems to have shown himself in the past a zealous and valuable officer should have suffered so severely for seeking to give effect to opinions which, however unwarranted in this or any other particular case, have without doubt some general basis of fact upon which to found their existence.

It is because we attach the highest importance to the maintenance of "lay" authority and control in our hospitals as the only safeguards against the spirit of professionalism, which, not wilfully, but by force of circumstance, would ruin them as philanthropic and religious institutions, that we reprobate the more strongly anything like precipitate and ill-advised action where no reasonable ground for complaint exists. The course pursued by the late secretary of the Royal Hospital for Diseases of the Chest has for the moment weakened the forces at command for the moral sanitation of our hospitals. He supped full of the detestable horrors provided by his counsellor and friend, and in the nightmare which followed he saw his own institution transformed to a torture house and its staff become a congregation of ghouls.

In dealing with subjects so delicate, we need, above all things, prudence. And prudence is exactly the quality which we seek for vainly in this quarrel. It is best attained by appraising the value of the interests in our keeping. The council never seem to have been particularly averse from the consequences of a rupture with their representative officer, and by their own showing withheld from him their confidence before they discontinued his services. The medical staff were simply fighting for their own hand—absolute independence—the goal they keep steadily in view, as the Russians, Constantinople; while the secretary appears to have entered with a light heart upon a crusade which should never have been undertaken without due reflection that though success may justify, to fail is criminal.

It is no small matter that something has been done to foster afresh in the minds of the patient class a suspicion that they are the subjects of experiments. The ghost of that fear is difficult to lay. It has stalked through thousands of ignorant minds, lending terror to suffering, and will haunt the neighbourhood of the City-road for many a month to come.

Nor is this belief altogether confined to a particular class. It is shared, as we have evidence in the Royal Hospital case, by a section of hospital supporters. And we think that upon the whole it is a right and healthy feeling which would set some limit to the independence of the medical staff, or at least demand some guarantee that the enthusiasm of science shall never weaken the sense of responsibility in respect of the welfare of each individual patient.

Those who really know what the work of our hospitals is will not need to be told that at the present day the tenderest care, coupled with the exercise of the utmost skill, are the portion of every sufferer. Once let the patient be admitted he is certain of advantages few can command in their own homes, and the charge of unnecessary or experimental operating may be dismissed as baseless. Over and over again in the experience of all our hospitals is an operation, in itself interesting, and possibly knowledge-laden, refused because the prospect of benefit to the subject is not sufficiently certain to justify it.

We should be sorry to believe that the physicians and surgeons of our great hospitals are not to be fully trusted to restrain themselves and also the younger and more ardent spirits who are desirous to emulate their seniors' doughty deeds, but nevertheless we claim that, if human life and limb are as sacred in our hospitals as elsewhere, it is because the humane considerations which animate our physicians and surgeons find conditions favourable to their development in the religious and philanthropic elements supplied by a lay administration. AGRICOLA.

